# Content validity testing of the INTERMED Self‐Assessment in a sample of adults with rheumatoid arthritis and rheumatology healthcare providers

**DOI:** 10.1111/hex.13978

**Published:** 2024-02-17

**Authors:** Kiran Dhiman, Marc Hall, Trafford Crump, Alison M. Hoens, Diane Lacaille, James A. Rankin, Karen L. Then, Glen Hazlewood, Cheryl Barnabe, Steven Katz, Jason Sutherland, Erika Dempsey, Claire E. H. Barber

**Affiliations:** ^1^ Department of Medicine, Cumming School of Medicine University of Calgary Calgary Alberta Canada; ^2^ Faculty of Nursing University of Calgary Calgary Alberta Canada; ^3^ Department of Community Health Sciences, Cumming School of Medicine University of Calgary Calgary Alberta Canada; ^4^ Department of Surgery, Cumming School of Medicine University of Calgary Calgary Alberta Canada; ^5^ Arthritis Research Canada Vancouver British Columbia Canada; ^6^ Department of Physical Therapy University of British Columbia Vancouver British Columbia Canada; ^7^ Arthritis Patient Advisory Board, Arthritis Research Canada Vancouver British Columbia Canada; ^8^ Department of Medicine University of British Columbia Vancouver British Columbia Canada; ^9^ McCaig Institute for Bone and Joint Health Calgary Alberta Canada; ^10^ Department of Medicine University of Alberta Edmonton Alberta Canada; ^11^ Centre for Health Services and Policy Research University of British Columbia Vancouver British Columbia Canada

**Keywords:** care complexity, outcomes research, qualitative research, rheumatoid arthritis

## Abstract

**Background:**

Care complexity can occur when patients experience health challenges simultaneously with social barriers including food and/or housing insecurity, lack of transportation or other factors that impact care and patient outcomes. People with rheumatoid arthritis (RA) may experience care complexity due to the chronicity of their condition and other biopsychosocial factors. There are few standardised instruments that measure care complexity and none that measure care complexity specifically in people with RA.

**Objectives:**

We assessed the content validity of the INTERMEDS Self‐Assessment (IMSA) instrument that measures care complexity with a sample of adults with RA and rheumatology healthcare providers (HCPs). Cognitive debriefing interviews utilising a reparative framework were conducted.

**Methods:**

Patient participants were recruited through two existing studies where participants agreed to be contacted about future studies. Study information was also shared through email blasts, posters and brochures at rheumatology clinic sites and trusted arthritis websites. Various rheumatology HCPs were recruited through email blasts, and divisional emails and announcements. Interviews were conducted with nine patients living with RA and five rheumatology HCPs.

**Results:**

Three main reparative themes were identified: (1) Lack of item clarity and standardisation including problems with item phrasing, inconsistency of the items and/or answer sets and noninclusive language; (2) item barrelling, where items asked about more than one issue, but only allowed a single answer choice; and (3) timeframes presented in the item or answer choices were either too long or too short, and did not fit the lived experiences of patients. Items predicting future healthcare needs were difficult to answer due to the episodic and fluctuating nature of RA.

**Conclusions:**

Despite international use of the IMSA to measure care complexity, patients with RA and rheumatology HCPs in our setting perceived that it did not have content validity for use in RA and that revision for use in this population under a reparative framework was unfeasible. Future instrument development requires an iterative cognitive debriefing and repair process with the population of interest in the early stages to ensure content validity and comprehension.

**Patient or Public Contribution:**

Patient and public contributions included both patient partners on the study team and people with RA who participated in the study. Patient partners were involved in study design, analysis and interpretation of the findings and manuscript preparation. Data analysis was structured according to emergent themes of the data that were grounded in patient perspectives and experiences.

## 1 INTRODUCTION

Rheumatoid arthritis (RA) is the most common autoimmune arthritis affecting approximately 1% of adults worldwide.^1^ The condition is characterised by joint inflammation leading to pain and progressive joint damage, if left untreated.^1^ Even when disease is ‘well controlled’ or in remission, individuals with RA may experience ongoing pain, fatigue and poor functional status.^2^ Furthermore, RA is associated with extra‐articular manifestations such as interstitial lung disease and comorbidities such as cardiovascular diseases and osteoporosis.^1^ There is also a high prevalence of mental health concerns such as depression and anxiety.^3^ Individuals with RA can be impacted by other social determinants of health such as employment status, food and housing insecurity and social considerations such as exclusion and discrimination, among others.^4^ These can contribute to lower quality of life, resulting in additional complexity in addressing healthcare needs.^5^

Defining the health status of patients has evolved over time. Previous focus was on number and types of comorbidities and number of medications.^6^, ^7^ Over time, the concept of care complexity has emerged, which considers both medical and nonmedical aspects of health. It describes the interplay between one or more chronic health conditions and possible mental health concerns, along with social determinants of health such as housing, food insecurity, lack of transportation, structural racism and other factors that negatively influence health or hinder access to healthcare.^8^ The INTERMED is a commonly used interview‐based instrument that measures care complexity across four biopsychosocial domains: biological, psychological, social and health systems across the past, present and future.^9^ The INTERMED was developed as a standardised way to measure complexity. It serves as a communimetric instrument to measure complexity, and also highlights domains to direct care in an interdisciplinary setting.^6^ The INTERMED can only be administered by a healthcare provider (HCP) who has completed specific training. The INTERMED was later adapted into a patient‐reported instrument called the INTERMED Self‐Assessment (IMSA).^10^

The INTERMED has been used in a wide variety of settings^11^, ^12^, ^13^; however, it can be challenging to administer.^10^ The interview itself is approximately 20 min in length,^12^ which may be unfeasible due to resource and time constraints.^10^, ^14^ More recently, the IMSA was developed to overcome these limitations^10^ and has undergone feasibility, face validity and reliability testing.^10^ Face validity was ascertained by asking patients the following questions: ‘Do you think that these questions were appropriate to ask?’ (yes/no) and ‘Did we miss issues pertinent to your care?’ (yes/no).^10^ No cognitive debriefing was conducted. Given the recency of its development, few study teams have tested how well the IMSA measures complexity.^13^, ^15^ Furthermore, to our knowledge, no studies exist on the psychometric properties of the IMSA in individuals living with RA.

Interdisciplinary care is considered the optimal treatment model for RA patients with higher care complexity. Interdisciplinary care teams may include family physicians, physiotherapists, occupational therapists, social workers, nurses and nurse practitioners, pharmacists and mental healthcare professionals, among others,^16^, ^17^ working together to promote holistic and patient‐centred care.^18^ However, interdisciplinary care delivery is costly and potentially difficult for certain patients to access as the rheumatology workforce is primarily located in urban centres.^19^ Rheumatology workforce deficits are expected globally in the coming decades,^20^ which will exacerbate accessibility of care over time. Allocating limited resources efficiently while supporting patient needs can be challenging. Unfortunately, there is no standardised method in rheumatology to measure which patients have greater care complexity and could benefit from interdisciplinary resources.^2^, ^21^

The objective of the present study was to test the content validity of the IMSA through cognitive debriefing interviews with a sample of patients with RA and rheumatology HCPs. The results of this study contribute to the body of knowledge around care complexity in RA. This work was planned as an initial step before local implementation of the IMSA. Measuring complexity can facilitate HCP teams in efficiently directing interdisciplinary care^10^ and improve patient ‘fit’ with the resources that they need at the right time.

## 2 METHODS

### 2.1 Study design

We conducted a series of cognitive debriefing interviews with patients diagnosed with and receiving care for RA and with rheumatology HCPs. Cognitive debriefing is a method to evaluate content validity, which includes the relevance, comprehensiveness and comprehensibility of the item stems and answer sets of a psychometric instrument.^22^, ^23^ Typically, cognitive debriefing is recommended to adapt an existing instrument to a specific population and ought to be conducted before further psychometric testing and/or item reduction.^24^ The aim of the cognitive debriefing interviews was to (1) assess whether the English‐language version of the IMSA^25^ (https://www.intermedconsortium.com/instrument/) was clear and understood as intended by patients with RA and by rheumatology HCPs; (2) determine any revisions required to enhance the relevance, understandability and comprehension of the item stems and answer sets; and (3) gather insight into specific topics relevant to care complexity in RA and that are not addressed in the IMSA. This project was approved by the Conjoint Health Research Ethics Board at the University of Calgary (REB20‐0422). All participants provided their consent to be involved in this project.

### 2.2 Study setting

This study was conducted in Alberta, Canada, and involved four rheumatology clinics across the province, including the Kaye Rheumatology Clinic in Edmonton and the Northeast Rheumatology Clinic and Associates Clinic in Calgary. The study primarily centred at the Richmond Road Diagnostic and Treatment Center Rheumatology Clinic in Calgary. It is the largest rheumatology clinic in Southern Alberta providing care to thousands of patients with rheumatic diseases yearly.

### 2.3 Eligibility and recruitment

#### 2.3.1 Patient participants

Participants were recruited through posters and brochures displayed within and distributed by HCPs from participating rheumatology clinics in Alberta. Postings about the study were available on trusted national websites, including Arthritis Society Canada and Arthritis Research Canada. Patients were also identified and contacted by email through two studies (Rheum4U and A Better Match) conducted at the University of Calgary. Rheum4U is a quality improvement and research platform in the Division of Rheumatology at the University of Calgary^26^ and A Better Match was a study of text‐based messaging to support patient care in RA.^27^ Patients in these two studies provided consent to be contacted about future research opportunities. From those who consented to be contacted, we invited adult participants (18 years of age and older) who had a diagnosis of RA and were not concurrently participating in another study. Patients who learned about the study by posters or brochures were instructed to contact the study coordinator using the contact information provided. All patient participants were sent an email containing information about the study and a link to an online consent form, with a short demographic survey and the IMSA following this. Fifty emails were sent on 25 October 2022 to Rheum4U participants. Reminders were sent 1 and 3 weeks after the initial email. Twenty‐one participants from A Better Match were contacted between 11 July and 15 July 2022. Once consented, an email requested the participants to indicate their preferred day, time and interview method (telephone or Zoom videoconferencing) and email reminders were sent 1 week later if a participant did not reply.

#### 2.3.2 Rheumatology HCPs

Rheumatology HCPs included any physician or healthcare professional involved in rheumatology care including rheumatologists, residents, nurses, pharmacists and physiotherapists. Rheumatology HCPs were initially recruited across Alberta through email announcements sent by the University of Calgary rheumatology divisional manager and announcements at divisional rounds. To broaden recruitment, a message was included in the Canadian Rheumatology Association email newsletter to reach providers nationwide. Due to the low recruitment of HCPs, we included some members of the study team. These team members were not involved in interview guide development, nor did they review the IMSA before the interview. These members were to be involved in future implementation of this instrument in our ambulatory care settings and their feedback was valuable as part of the planning process. Furthermore, no members of the research team had any involvement in the development of the IMSA. Rheumatology HCPs, if interested in participating, were instructed to contact the study coordinator and received a link to an online consent form and a short demographic survey followed. If the consent form and survey were not completed, an email reminder was sent 1 week later. Once consented, an email requested participants to indicate their preferred day, time and interview method (telephone or Zoom videoconferencing) and email reminders were sent 1 week later if a participant did not reply.

### 2.4 Procedure

After consenting to the study, patient–participants responded to demographic questions (year of birth, year of RA diagnosis, gender identity, highest level of education attained and race/ethnicity) and completed the IMSA. For HCPs, brief demographic information was collected (HCP type, years of practice, sex at birth and gender identity).

A single semi‐structured interview guide was developed by two members of the research team (C. E. H. B. and K. D.) for both patients and HCPs (Supporting Information S1: Supplement S1). Questions in the interview guide focused on comprehension of the item and answer set, confidence/recall for providing a response, relevance of the item, questions related to specific items in the IMSA and concluding questions that asked participants about the comprehensiveness of the IMSA and what care complexity challenges exist in RA that may be missing in the instrument. Two researchers trained in qualitative methods (K. D. and M. H.) conducted the interviews over Zoom through videoconferencing or by telephone using the cognitive debriefing semi‐structured interview guide.

The interviewer administered each of the IMSA items one at a time verbally and encouraged participants to ‘think aloud’ when interpreting the items and providing responses. Participants were asked probing questions about their comprehension of survey items, how they arrived at answer choices (recall), appropriateness of the answer choices provided (response) and their perceived relevance of the item to their health care. When participants encountered difficulties with the items, the interviewers asked for suggestions on how to modify the item and the answer set. Interviews were audio‐recorded and transcribed verbatim by a transcription service. Transcripts were checked for accuracy by the study coordinator and identifying details were removed. By way of appreciation, participants received a $50 gift card.

### 2.5 Analysis

Transcript analysis was guided by Willis'^23^ and Wills'^28^ protocols for analysing cognitive interview results. Analysis focused on a reparative framework,^28^ which involved ‘finding and fixing’ participant‐identified issues with the instrument.^28^ Two coders (K. D. and M. H.) independently analysed the transcripts: K. D. by IMSA item and M. H. by participant. Results were compiled across interviews and organised by IMSA item. Coders then independently created a text summary of the results for each item, noting findings for comprehension, recall, response and relevance. The coders' results were compared, and differences in text summaries and recommendations were discussed until consensus was reached in a smaller working group (K. D., M. H., C. E. H. B.). Recruitment continued until saturation occurred, which was reached after only a few interviews with both patient participants and HCPs. Saturation was defined by the recurrence and repetition of themes by both groups of participants. Results were presented to the broader research team and patient partners. Due to the volume and depth of repairs recommended by participants, consensus was reached to halt further interviews. This approach parallels Willis,^29^ where if major problems are discovered with an instrument, there is little to no benefit to continue until repairs are made.

## 3 RESULTS

Seventeen people with RA completed the consent form for the study. Nine people participated in the interview and eight people did not respond to the interview day/time/method request email. One participant partially completed the items on demographics (gender identity, year of RA diagnosis and IMSA only) and eight participants fully completed the items (Table 1). The median (Q1, Q3) age was 59.5 years (56, 62) and the median number of years lived with RA was 5 (3, 6). Eight of the nine participants identified as female and all participants identified as White/European. Most participants either had a high school diploma or equivalent or a college/nonuniversity certificate or diploma. The IMSA cut‐off score for detecting complexity is 19^10^; the median score for the patients was 15 (12, 22). Three patients were deemed of higher complexity using this cut‐off score of complexity. Our sample compares similarly to other Canadian RA cohorts in terms of age, years lived with RA, education and ethnicity/race.^30^

**Table 1:** Demographic characteristics of patients who participated in IMSA cognitive debriefing interviews.

Characteristics (*n* = 9)	*n*
Gender identity^a^	
Female	8
Male	1
IMSA complexity score (Range)	7, 26
Age (years), median (IQR)^b^	59.5 (9.5)
Participants aged 15–64 below, above IMSA complexity cut‐off^c^	4, 2
Participants aged 65+ below, above IMSA complexity cut‐off	1, 1
RA duration (Years), median (IQR)	5 (3)
Race/ethnicity^d^	
White	8
Indigenous (First Nations/Metis/Inuit)	1
Highest level of education^e^	
High school diploma or equivalent	2
Apprenticeship or trades certificate or diploma	1
College of another nonuniversity certificate or diploma	3
University certificate or diploma	1
Doctorate degree or equivalent	1

*Note*: Data presented are *n* unless otherwise specified.

Abbreviations: IMSA, INTERMED Self‐Assessment; IQR, interquartile range; RA, rheumatoid arthritis.

^a^ Gender identity categories (*n* = 0) not displayed: ‘Nonbinary or other gender identity’ and ‘Prefer not to answer’.

^b^ Age (*n* = 8).

^c^ The IMSA cut‐off score for detecting complexity is 19.

^d^ Race/ethnicity, *n* = 8 all participants identifying as White/European with one participant additionally identifying as Indigenous (First Nation/Metis/Inuit).

^e^ Highest level of education (*n* = 8).

Six rheumatology HCPs completed the consent form for the study. Five persons participated in the interview and one person did not respond to the interview day/time/method request email. Of these, three were rheumatologists, one was a rheumatology resident and one was a rheumatology clinic pharmacist. Three out of the five participants were female and had between 2 and 10 years of practice as an HCP. Two of the five HCP participants were study team members.

Participant quotes were organised into reparative themes based on their nature and recurrence across the IMSA items: (1) lack of clarity and standardisation with subthemes around phrasing of items and answer sets, language regarding mental health and social roles and inconsistency across answer sets; (2) item barrelling, where items contained multiple clauses simultaneously; and (3) timeframes, such as recall period or prediction of future events. The IMSA items were then organised by these themes to develop an overview of the possible modifications to the IMSA based on the reparative framework. Most items fit under a single theme, and some items had several issues relevant to a single theme or multiple themes (Table 2). Concluding thoughts about the comprehensiveness of the IMSA are also presented.

**Table 2:** Overview of reparative themes within the IMSA items.

	Lack of clarity and standardisation			Item barrelling	Timeframes
Themes and subthemes IMSA item^a^	Phrasing of items and answer choices	Language regarding mental health and social roles	Inconsistency across answer sets		
1a	✓				✓
1b	✓				
2	✓				✓
3					
4a	✓				
4b	✓				
5			✓		✓
6	✓	✓			✓
7	✓				
8	✓	✓	✓		
9a	✓				
9b	✓	✓			
9c	✓				
10	✓		✓		
11	✓			✓	
12	✓		✓	✓	
13	✓			✓	
14	✓		✓		
15	✓	✓			
16	✓				
17	✓		✓		✓
18	✓	✓			✓
19	✓		✓		✓
20	✓		✓		✓

Abbreviation: IMSA, INTERMED Self‐Assessment.

^a^ Refer to Supporting Information S1: Supplement 2 for items and answer sets.

**Figure 1:**
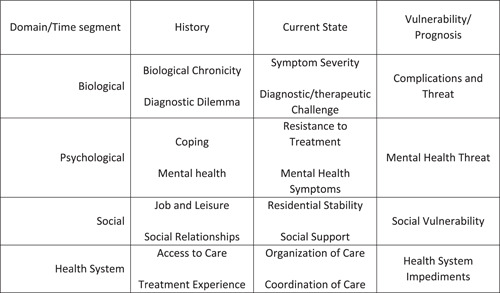
Question topics in the INTERMED Self‐Assessment according to domain and time segment.

Examples of how the IMSA items aligned with the three themes are presented below with participant quotes, with further examples shown in Table 3. Following this are remarks regarding the comprehensiveness of the IMSA. A table of IMSA items and answer sets along with participant‐recommended repairs to them are presented in Supporting Information S1: Supplement 2. Figure 1 provides an overview of the IMSA item topics in relation to their domains and across their time segments.

**Table 3:** Examples of reparative themes identified within IMSA items with supporting participant quotes.

IMSA item^a^	Participant quote(s)
*Reparative subtheme: Phrasing of items and answer choices*	
Item 1a	‘That was another one I wrote down, “Very badly worded”, because you can take this [item] in many aspects. It says “physical problems”… so for instance, I think it was … [during] COVID … I broke my wrist … so do you mean physical problems outside of RA or within RA? … RA constantly changes. You constantly have different flares and stuff. So again, that's just … It's just like it's way too big of a question with not concrete enough answers’. (Patient participant #19, female, IMSA score 9, age 55–64)
	‘… I suppose this one … I did have to think about that for a bit … how it's worded and … when I did have some physical problems … so I think this one was a tough one for me to answer and just because the rheumatoid diagnosis was quite new. Um, I even forget how I answered it, if I'm being honest … I think this one was a tricky one for me’. (Patient participant #21, female, IMSA score 14, age 34–44)
Item 4b	‘… I was thinking about the medication … any life changes. Oh … losing weight, diet … physiotherapy suggestions about, Oh, you know maybe you could wear a knee brace? Or, maybe you should get some hiking poles? Or, like all these other aspects, so it wasn't just … the medication and the appointments. It was all the other parts of it along the way, … to me … are just as important … so … I thought about all those different things’. (Patient participant #5, female, IMSA Score 12, age 65+)
	‘I don't know if I like the word, “appropriate”. So you can be on appropriate treatment, but that doesn't mean it's effective treatment. If I were a patient, I'd probably using the terms interchangeably for this question … but I don't think they're the same thing, right? You could be on totally appropriate treatment, and it just isn't working …. which … in rheumatoid arthritis population … it's unfortunately common and that's when we change things up … but that doesn't mean it was inappropriate’. (HCP Participant #1, male, rheumatologist, >10–20 years practicing)
Item 16	‘… I might add … “communicate and work well together”. [to one of the answer choices, and] “My doctors and health care providers do not work together … leading to problems every now and then”, [and] I would take out the “quite well”. And then just do, “Do not work together, leading to problems every now and then”, or problems or miscommunication or‐ … confusion. Yeah, then, “My doctors and health care providers do not work together”. And … then … “My doctors and health care providers do not work well together”, for my … like, “in coordinating my care”, something like that … I would add context to things, like, “cause you've got”, “I do not receive care, or my providers … provided by just one doctor”. I would just add context to the last one, that they should be working together or something’. (HCP Participant #3, female, rheumatologist, >2–10 years practicing)
	‘… Who, which doctors are you talking about? Which problem? And … what do you mean by work together well? Because often in rheumatology, the family doctors defer any decision about the rheumatoid arthritis to the rheumatologist … I'm not gonna speak to the family doctor every time they make a medication change …’ (HCP Participant #4, male, rheumatologist, >2–10 years practicing)
*Reparative subtheme: Language regarding mental health and social roles*	
Item 8	‘I think … there's a lot of … awareness … mental health is more out in the public sphere now … so I think … “mental health concerns” as opposed to psychological problems [as written in the item stem] … [and] … something like, “do you have any concerns related to your mental health such as being tense, anxious?” [rephrasing the item stem]. [I] … don't know if we still use … down and blue as a … descriptor … for low mood. I think you could … actually say low mood … then “confused” doesn't fit there for me. Yeah … seems odd … to have … I was trying to … think about … unmotivated or … some other … symptoms of … depression … I think would be, would be more … helpful there … and so I guess in terms of the responses, if … we can remove the word ‘[mental health] problems’. (HCP Participant #2, female, pharmacist, >2–10 years practicing)
Item 9a	‘… If they don't answer … maybe they can give you a reason why … because then you know … why you're upsetting them and, or is there a better way for us to ask this question? … You're trying to get basic information from people that they're comfortable to answer and you don't want them to fudge the answer because you don't want them to lie because they feel it's too invasive. What are you trying to find out by asking if somebody has a job? … What do you actually wanna know? It's a bit, it's a bit vague and maybe people feel put off by that question too’. (Patient participant #10, female, IMSA score 18, age 55–64)
Item 15	[Talking aloud and mentioning possible revisions to the item and answer set] “And then one or more specialists for my mental health [instead of ‘mental problems’ as written]”. Um, so social workers can be specialists use for mental health. So the‐ … the separation there would be, um … Yeah, uh, so what this is all health related, so it's not going to be financial support that that person is providing. So, um‐ … you could see putting that in with mental health maybe. Just that current, current lingo’. (HCP Participant #2, female, pharmacist, >2–10 years practicing)
*Reparative subtheme: Inconsistency across answer sets*	
Item 2	‘… I suppose I was a little, a little confused by some of the answers … because … I guess with rheumatoid arthritis, specifically … I … thought …. the origin was always unknown, I guess’. (Patient Participant #21, female, IMSA score 14, age 35–44)
	‘… The other thing I'd say is, I don't know if most patients would know what … “routine investigations” [means]. But I think … your next [answer choice], “after a lot of investigations”, that does kind of help clarify it … what I think is a lot, I don't know if someone else might think is a lot’. (HCP Participant #3, female, rheumatologist, >2–10 years practicing)
Item 17	‘… The nuances between answers two, three and four, I'm not sure … how well people would differentiate on those three, and how I would interpret it … I'll ask patients how do they think they're doing, like, after starting medication, and even asking them to tell me do they think they're 50% better or 80% better … And I find people are much more able to communicate that’. (HCP Participant #3, female, rheumatologist, >2–10 years practicing)
	‘… For the second part [answer choice B], “I expect only slightly, or, slight worsening of my physical health”. It depends, slight worsening, is it more than 50% worsening, or just 10, 20% worsening? So sometimes people's expectations are different, and their tolerance of the symptoms are different. So maybe a little bit of more quantification of what that's like. Could be more than 50%, less than 50% helps to answer the question’. (HCP Participant #5, female, Rheumatology resident, practicing 2 years or less)
Item 19	‘Well, the question is saying, in the next six months, do you expect a change? And then, the three options [answer choices] all say in the next six months. And then the last one goes, needed immediately … Maybe that's not an issue … The other thing is … what if I am living at home and getting home care already?’ (HCP Participant #4, male, rheumatologist, >2–10 years practicing)
	‘I would say if I was the patient, I'd say, “I don't know”. Because it's asking the patient to predict something, that … not the patient nor the physician knows what's going to happen. The last answer, it changed to “another living situation is needed immediately”. … so if that's the situation, they're having problem right now … that needs to be addressed. But other changes in the next six months, that's a little hard to predict. I'm truly not sure what this question is asking about, and what would be the purpose of this question’. (HCP Participant #5, female, rheumatology resident, practicing 2 years or less)
*Reparative theme: Item barrelling* b	
Item 11	‘… I guess, “immediate adjustments are needed” [one of the response choices] … I don't know if that covers I need to move somewhere else … it's the closest thing to … you know, if somebody's in a situation … I have a friend who just got a knee replaced and getting up and down the steps to the last few appointments were very impossible, and painful, and crazy and dangerous … so if somebody needs to move urgently. Um, I don't know if it would be … immediate adjustments are needed is a strong answer … I don't know if … adding a fifth one to say I need to move now’. (Patient Participant #20, female, IMSA score 7, age 55–64)
Item 12	‘So maybe they need to add on [an answer choice], “I'm not in need of assistance but have help if needed”, right? Because maybe you've got that little old lady by herself in a house and she's independent, but what if you need something?’ (Patient Participant #19, female, IMSA score 9, age 55–64)
	‘I jumped around between [answer choices] one and two. Like, okay, I don't think I need any assistance right now, but when I did, then it was available. Anyway. So, um, yeah. So, yeah, maybe three as well. So, I … yeah, it is maybe one, two, three. Like, I could answer any of those (laughs)’. (Patient Participant #20, female, IMSA score 7, age 55–64)
*Reparative theme: Timeframes*	
Item 1a	‘Well, that three‐month period [mentioned in the answer choices] … for myself … yes, I experience problems … not necessarily for short periods of time, but for longer periods of time … I just feel like the three months aspect [in the answer choices] over that five years [in the item stem] is kind of putting you in a box … it's limiting your answer. Like when I answered it, I answered it, but always with a “but”’. (Patient Participant #23, female, IMSA score 25, age 65+)
	‘Well, our population is rheumatoid arthritis. Seems to me that the vast majority would experience physical problems the past five years. So I think … it's not so much a yes or no … but more so being marker of potential disease activity … if that's how you're using it, whether … those timeframes are appropriate or not, I'd have to think about’. (HCP Participant #1, male, rheumatologist, >10–20 years practicing)
Item 6	‘Sometimes, the problem I have is that, it's like that one question before … there's … a two‐edged answer in some ways, because some of the problems are still affecting me … they influenced my daily life, but I don't know that about the long lasting effect [answer choice wording]. I think it's a funny question…when they're putting it in the past … what did they mean by in the past?… A year, 10 years, you know, 50 years, or what, you know?’ (Patient Participant #23, female, IMSA score 25, age 65+)
	‘… If I'm trying to think of how it would help me in the visit … I'm not … sure it would help me, because when, in the past?… If somebody's currently … struggling with this, then yes, that's really helpful … if somebody had this ten years ago and is doing well right now, I don't need to get into it with them, right?’ (HCP Participant #4, male, rheumatologist, >2–10 years practicing)
Item 18	‘Yeah, it was just … thinking about what's coming up for me in the next six months, and what that's going to mean for my mental health … I still think a response for this question that would be good is “I'm not sure”… for a lot of people, they can't predict that as well … I still think like a three month could be better because I just feel like for a lot of people, it's easier for them to imagine where they're going to be in three months rather than half a year’. (Patient Participant #18, female, IMSA score 26, age 15–24)

*Note*: Some items that appear here may have been presented in the results section under a specific reparative theme, but are presented here under a different reparative theme; Table 2 demonstrates the multiple reparative themes that appeared across IMSA item.

Abbreviations: HCP, health care professional; IMSA, INTERMED Self‐Assessment.

^a^ Refer to Supporting Information S1: Supplement 2 for items and answer sets.

^b^ Two items are presented here as the item barrelling theme appeared across three IMSA items, with one of the items within this reparative theme already presented in the Section 3.

### 3.1 Lack of clarity and standardisation

#### 3.1.1 Phrasing of items and answer sets

Clarity and wording concerns made up a significant portion of the results. Participants perceived that items and answer sets were sometimes poorly worded, and the intention of the items was unclear. Due to the lack of clarity, it was difficult for some patients to select an answer to an item that best fit their situation. For example, Item 4A is, ‘Do you think your doctors understand the origin of your current physical problem/s?’. It was unclear to patients and HCPs what the term ‘origin’ implied.

Oh, this one confused me a little bit to tell you the truth … speaking to like, with my physical problem … I mean … with RA … I'm not sure anybody knows what causes it … so … it doesn't take them long to, you know … diagnose the problem, and I've been treated ever since. But did they actually understand the origin … of what caused it? Is that what you're asking? (Patient Participant #1, male, IMSA score 15, age unknown)

I don't know what origin means. So does origin mean diagnosis, or does origin mean why I have this diagnosis? So in other words … my doctor is clear that I have rheumatoid arthritis, there's no doubt, or my doctor doesn't know why I have rheumatoid arthritis? (HCP Participant #1, male, rheumatologist, >10–20 years practicing)

Another example involved Item 11: ‘Is your home living situation satisfactory? Or are adjustments needed, such as home modifications, receiving home care, or going to live somewhere else?’. Some patients were unclear as to how satisfaction with their home living situation should be interpreted:

… So when I was reading this question … it made me think … how is … your emotional living at home … but then I … finishing the question, I realized that it was about like physical monitor‐ modifications that can like help you move around the house. Yeah, but the first part of the question to me sounded more like … do you have like a safe, comfortable home? Rather than … is it accessible for you? (Patient Participant #18, female, IMSA score 26, age 15–24)

Um, I don't know. I think home modifications would be readily interpretable. Um, I was just thinking about like … I don't know. Yeah, I'm trying to think about, even like, like the question structure. (HCP Participant #2, female, pharmacist, >2–10 years practicing)

#### 3.1.2 Language regarding mental health and social roles

Other findings regarding item wording related to language describing mental health and social roles. Several IMSA items and/or answer sets refer to ‘psychological problems’, where participants commented on changing the phrase to ‘mental health’ to limit possible stigmatisation, as mentioned below:

… I don't think people will respond well to ‘psychological problems’… I think there's a lot of … awareness … mental health is more out in the public sphere now. And so I think … mental health concerns as opposed to psychological problems, but I think also … we all have mental health. (HCP Participant #2, female, pharmacist, >2–10 years practicing)

Item 9B contains an answer choice that reads, ‘I am a housewife taking care for the household and others’, referring to a caregiving role that is gendered. Participants suggested rephrasing this to be neutral and inclusive of people performing a caregiving role, regardless of sex or gender, as one of our participants articulated their ideas about a possible revision to the answer choice:

So I like ‘do you work outside of the home’, or ‘do you have paid employment’, kind of language. So, um, not wanting to degrade work that happens inside the home. Um, obviously, the housewife, um‐ Yeah. So, language for that … ‘I am providing care for, you know, children and/or others in the, in the home’ or whatever. Yeah, something like that. (HCP Participant #2, female, pharmacist, >2–10 years practicing)

#### 3.1.3 Inconsistency across answer sets

The issue of inconsistency across answer sets appeared often. Throughout the IMSA, answer choices would follow a particular pattern and then include one or more answer choices deviating from the pattern. For example, Item 5 is ‘In the past 5 years, how did you cope with stressful, difficult situations?’ and answer choices B and C (Supporting Information S1: Supplement 2) refer to difficulty coping in these situations, leading to tensions with partners, family or others. However, tension with others was not mentioned in all answer choices. Answer choice D refers to always experiencing difficulties with stressful and difficult situations, making a person upset and tense, which deviates from the pattern where the other answer choices describe tensions with others. This was acknowledged by two HCPs, who mention that the answer sets do not follow a similar structure across items in the IMSA overall, and how Item 5's answer set in particular followed a structure that was different from what they were expecting, as outlined by the quotes below:

… it's a little bit weird how the [answer choices] don't follow the same kind of syntax or structure. Like, um, the middle two talk about the result of them … and then two [answer choice B] says, ‘Often led to tensions and problems’. And then in three [answer choice C] … I would be expecting it to say, ‘I always experience difficulties with stressful, difficult situations that lead to tensions and problems with my partner, family or other people’. Or something like that. (HCP Participant #4, male, rheumatologist, >2–10 years practicing)

So, like, ‘sometimes I have difficulties with coping with stressful difficult situations’, okay, fair. But why does that, by definition, at‐ as this answer seems to suggest, that means that it results in tension, problems with partner, family or other people? So if you are someone who … feels sometimes I have difficulty coping with stressful, difficult situations, but does not sometimes result in tension, problems with my partner, family or other people. Now what‐ how would you answer the question now? (HCP Participant #1, male, rheumatologist, >10–20 years practicing)

This recurred in Item 10, which is, ‘How do you generally relate to other people?’, in which answer choice B refers to experiencing tensions with others and answer choice D refers to contacts and friendships deteriorating into quarrels and conflicts (Supporting Information S1: Supplement 2). Tensions, conflicts and quarrels were not mentioned in any of the other response options. Confusion around the rationale for these types of responses and the inconsistencies within the answer set were noted by two patient participants:

Uh, some of the answers … I thought were a little strange …. I was wondering kind of why it kept going to the tense or the quarrels and conflicts …. Like I wasn't quite sure about that, I suppose…I guess I didn't really know why it was going to the, um, to the sort of the angrier side of things. (Patient Participant #21, female, IMSA score 14, age range 35–44)

I answered this one, the first [answer choice] … I've got lots of friends and I socialize well. As it got further in, I thought, ‘Oh, isn't this interesting? Where are they going with this?’. But I didn't understand what's the point of that …. I was surprised as … it was … more negative stuff. (Patient Participant #10, female, IMSA score 18, age range 55–64)

#### 3.1.4 Item barrelling

Item barrelling refers to multiple clauses being included in an item, but only allowing for one answer choice. Some items were double, triple or even quadruple barrelled, which made them challenging to answer. Barrelling also made it difficult for researchers to know which parts of the item a patient's response applied to. A prominent example was Item 13, which asks, ‘Do you experience problems in getting the care you need due to living too far away, not having any insurance, or not speaking the language very well, or differences in culture?’. Participants, while thinking aloud, would answer each part of the item presented individually or provided ways to present this question differently as demonstrated by the quotes below:

… They [the items in the question stem], they're not problems for me … because I live in the city. We have healthcare. English is our first language. Um, culture, I've been here 32 years, I guess, so (laughs) I think culture is, um, is normal to me now. (Patient Participant #20, female, IMSA score 7, age range 55–64)

Too much … of a question. Yeah … You can separate these out into one, two, three, four questions and have … the various answers for each one of them or you could have … a tick box like select all that apply … type of thing … if this is an electronic survey like display logic for, for digging into … how often something happens. (HCP Participant #2, female, pharmacist, >2–10 years practicing)

#### 3.1.5 Timeframes

Some items asked patients to recall health information from years or months ago. This caused challenges for patients answering the item and HCPs commented on the relevance of the timeframes for people with RA. For example, Item 2 is ‘How difficult has it been in the past 5 years to diagnose the physical problems you experienced?’, with participants stating the following:

… I was going back even farther [than 5 years] and then … I realized, ‘Well, you know what? This is five years … I'm just gonna go with the way it went down when I was actually sent along to [Rheumatologist Name]. So how difficult has it been in the past five years? [Restated the question]. Well, it's actually been six, so I just kind of went with that … I thought, Well, we need to go back to when this all started … that's a long time. But for me it was longer even (laughs). (Patient Participant #5, female, IMSA score, 12, age 65+)

Um, y‐ like, you could shorten the interval, but I think, like, a lot of our patients do … have had issues for five years. I'd probably say two years would be sort of more acute and relevant, but … if you're looking at the patients who are more complex and grumbled before they got to us, then five is probably more appropriate. But you could shorten it to two. (HCP Participant #3, female, rheumatologist, >2–10 years practicing)

Other items asked patients to predict future physical and mental health challenges and use of supports in the next 6 months, which both patients and HCPs found challenging. Predicting future health needs was further complicated by the nature of RA, where patients describe RA as being unpredictable. Item 17 is ‘In the next 6 months, do you expect your physical health to change?’, with patients responding with the following:

I have no clue, and I feel like my doctors have no clue either. So I, I wasn't sure how to answer it. I, I felt like that was like, I don't know, a hard thing. And I'm also like going to be changing medications and like doing new things. So I don't know, those might work. They might not. (Patient Participant #18, female, IMSA score 26, age 15–24)

… It sometimes takes us six months to diagnose someone. So not everyone would feel anything different in six months. But at the same time, it's hard to ask a patient, ‘How do you think about next year, or next two years?’ So I think from that perspective, it's fair to ask six months, but not necessarily. I think, that's going to change the whole picture of the patient management. (HCP Participant #5, female, rheumatology resident, practicing 2 years or less)

Some items had several issues as exemplified by Item 20, which is ‘In the next 6 months, do you expect that you will be in need of more help and support?’. In addition to the challenge in predicting future needs, what patients define as ‘help and support’ and what patient expectations might be were perceived as vague:

… it was kind of more like, help and support with what? Because … I feel … in certain areas of my life, I will need more … help and support. But … when it comes to my … physical health, again, I just don't know. So I think … emotionally … when it comes to like therapy and counseling and stuff like that, I think I will be in need of more of that. But again, with my physical health, I just don't know what I'll need. (Patient Participant #18, female, IMSA score 26, age 15–24)

What they're [patients] expecting, based on what they presented to us at this point? Or what they're expecting based on what the diagnosis is? Or what their hopes are? So it's lots of things that you can think about, which it's hard to predict. I would take away the whole question. (HCP Participant #5, female, rheumatology resident, practicing 2 years or less)

#### 3.1.6 Comprehensiveness of the IMSA

Finally, when asked about elements of complexity that may be missing from the IMSA, most participants commented that the instrument was comprehensive in addressing potential elements relating to their care. One patient participant mentioned how their plans for retirement shifted significantly due to their diagnosis and suggested an element regarding future planning or life transitions. From an HCP perspective, one individual suggested adding question/s regarding prior and/or current social supports. This was so that physicians or other HCPs can plan for and/or provide resources for the patient, depending on their situation.

Revisions were deemed necessary to all but one item in the IMSA. Due to the number and scope of repairs required to amend the IMSA for use in RA, it was unfeasible to revise it using a reparative framework, as the resulting instrument would no longer retain its measurement scheme.

## 4 DISCUSSION

To our knowledge, our study is the first to assess content validity by performing cognitive debriefing of the IMSA with RA patients and rheumatology HCPs. Overall, the IMSA was perceived to be comprehensive in terms of complexity domains. However, participants pointed out several examples that demonstrated that the instrument did not meet key elements necessary for content validity for use in RA. This was primarily due to issues around item relevance, comprehension and recall periods used for the items. This impression was shared by both patients and HCPs in our sample, with similar themes identified by both groups of participants.

The INTERMED and IMSA^4^ have been used in various populations such as primary care,^31^ chronic pain,^13^ orthopaedics^32^ and mental healthcare settings.^11^ The IMSA has been translated into several languages,^33^ including French, German, Dutch, Spanish and Japanese, and a version for older people, INTERMED for the Elderly (IM‐E), has also been developed.^34^ One example of the IMSA being used to allocate resources is a study performed in Switzerland that compared the IMSA to general practitioner (GP) opinion in predicting which patients would benefit from case manager intervention in primary care due to care complexity.^35^ This study had 331 patients complete the English version of the IMSA,^25^ then researchers asked GPs for their opinion on that same patient being assigned a case manager, with four possible answers varying on yes or no, and the usefulness of being assigned a case manager. Researchers found that three items from the IMSA were sufficient to predict GP opinions about patients benefiting from case management (Q1 A and B [presence of physical problems and presence of a long‐lasting chronic condition], Q3 [Physical limitations in performing daily activities] and Q9 A, B and C (employment status and engagement in activities/hobbies/volunteering]). Given the simplicity and brevity of a three‐item IMSA, researchers commented that this can serve as an ideal prescreening instrument and information could be readily available in electronic medical records, possibly eliminating questionnaire burden on patients. These three items only focus on biological and social aspects and may exclude other salient considerations in determining care complexity. The study also centred on GP perceptions of care complexity, which may differ from care complexity for rheumatologic conditions, as study authors note that complexity is context‐dependent.^35^ These differences could affect the use and relevance of instruments that attempt to measure complexity.^35^

In rheumatology, the use of care complexity measurement instruments has been scant. One of the few studies in this area is Koch et al.,^5^ where researchers used the INTERMED to identify patients who experience care complexity and correlate this with measures of healthcare utilisation and disease activity (including the Health Assessment Questionnaire, Short Form Survey 36‐item (SF‐36), Rheumatoid Arthritis Disease Activity Index, radiologic erosion scores and disease activity score‐28).^5^ Patients who scored high on the INTERMED (indicating care complexity) scored particularly high in the psychological domain of the INTERMED and were more likely to receive disability compensation. These patients also had increased healthcare utilisation in terms of emergency room visits, hospitalisations and specialist visits, even though they did not differ from ‘noncomplex’ patients in terms of disease activity. Patients who had care complexity also reported a worse global assessment of disease compared to physician global assessment of disease and scored worse on the SF‐36 on the domains of general health and vitality. While the INTERMED was effective at detecting care complexity, it is an interview‐based method that can allow for items to be clarified and explained if ambiguous, which may impact patient responses and influence their overall score.

While the INTERMED and IMSA measure important domains, they do not address RA‐specific domains. Authors of Koch et al.^5^ found that several patient‐reported surveys were required to supplement the INTERMED to determine the areas in which care complexity existed, which may be unfeasible for both patients and HCPs due to time constraints in clinical settings.^10^, ^14^ An ongoing literature review being conducted by our study team is investigating care complexity factors in RA and their impact on processes of care and/or RA outcomes. While some of these factors are captured in the IMSA including mental health, coping and housing instability, other complexity factors more pertinent to RA, such as functional status and disability,^36^ concordance with treatment plan^37^ and social support,^38^ were asked in a way that made it difficult for patients to understand and relate to their own experiences. These may uniquely drive complexity in RA and incorporating these domains into a tailored instrument may measure complexity more effectively in this population.

In addition to the lack of complexity measures and their specificity, there is a paucity of data regarding RA and care complexity generally.^4^ The available evidence suggests that RA is highly impacted by biopsychosocial factors. For example, race and intersectional inequalities can increase the risk of developing RA and these factors can sustain poor disease control through biological and nonbiological factors such as inequitable access to care and underrepresentation in research where disease outcomes in diverse populations are not well understood.^4^, ^39^, ^40^, ^41^

Furthermore, social determinants of health can potentially reinforce one another. Income inequality plays a causal role in poor health outcomes for various reasons^42^ and can contribute to disparities such as food insecurity.^43^ Working‐age adults who are food‐insecure have an elevated risk of developing chronic health conditions such as arthritis.^42^ People with RA who experience food insecurity have higher odds of depression and the odds increase when food insecurity increases in severity.^44^ People with arthritis who are food‐insecure are also at risk for medication underuse.^43^, ^45^ This is particularly concerning as pharmaceutical treatment of RA is critical to controlling disease activity and medication costs have risen dramatically due to the availability of biologics and targeted synthetic disease‐modifying antirheumatic drugs.^46^

Unfortunately, people who have overlapping and complex health and social needs are often met with uncoordinated and inefficient care, cycling through multiple providers and/or systems, with little benefit from these interactions.^8^ The current standard of care in RA involves routine investigations that primarily measure biological aspects including disease activity, functional status, comorbidities and medication use.^5^ Mental health concerns in people with RA are common, but there is inconsistent screening of these conditions during clinic visits.^47^ Beyond these measures, other social determinants of health and/or elements of complexity that can significantly impact RA outcomes are rarely considered.^5^ Measuring these needs would improve the knowledge that we have about care complexity in RA and allow for efficient use of resources to provide high‐quality and accessible care to improve health outcomes.^8^ A framework for understanding care complexity in RA and its impact on care processes and patient outcomes will be presented in future work.

There were some limitations to this study. Most HCPs interviewed were rheumatologists, with limited representation of other healthcare professions involved in rheumatological care. Two study team members were interviewed; however, their perceptions of the IMSA did not differ significantly from patient participants or the rest of the HCP participants. There was also little diversity in patients with RA who were interviewed in terms of age and ethnicity, as saturation was reached early. Furthermore, patients who actively seek care, participate in research studies and/or review rheumatology‐related websites were recruited, and our sample may be less representative of people with higher care complexity. The online English‐language version of the IMSA was used in this study and it is unclear if other studies or research teams revised the instrument to address the issues that our study found.

In conclusion, the IMSA did not have content validity within our sample of adults with RA and rheumatology HCPs. Due to the significant number of repairs and revisions suggested by participants, these would have changed the instrument significantly from its validated form, necessitating a change to its scoring and interpretation. The creation of a new complexity instrument in RA and/or exploration of other instruments that measure care complexity are needed that are relevant and appropriate for use in RA to better allocate resources to patients and improve health outcomes.

Future instrument development in this area should involve cognitive debriefing alongside repairs in an iterative fashion. Doing this early on in instrument development can help ensure inclusive language, clarity and consistency of item and answer choices anchors and appropriate length of the instrument.

## AUTHOR CONTRIBUTIONS

**Kiran Dhiman**: Investigation; methodology; project administration; writing—original draft; writing—review and editing; formal analysis. **Marc Hall**: Investigation; writing—review and editing; formal analysis. **Trafford Crump**: Conceptualisation; funding acquisition; writing—review and editing. **Diane Lacaille**: Conceptualisation; funding acquisition; writing—review and editing. **Glen Hazlewood**: Conceptualisation; funding acquisition; writing—review and editing. **Cheryl Barnabe**: Conceptualisation; funding acquisition; writing—review and editing. **Steven Katz**: Conceptualisation; funding acquisition; writing—review and editing. **Jason Sutherland**: Conceptualisation; funding acquisition; writing—review and editing. **Erika Dempsey**: Conceptualisation; funding acquisition; writing—review and editing.

## CONFLICT OF INTEREST STATEMENT

The authors declare no conflicts of interest.

## Supporting information

Supporting information.

## Data Availability

The authors confirm that the data supporting the findings of this study are available within the article and its Supporting Information materials.
