# Supplementary material for: Content validity testing of the INTERMED Self‐Assessment in a sample of adults with rheumatoid arthritis and rheumatology healthcare providers
Source: Health Expect. 2024 Feb 17;27(1):e13978. doi: 10.1111/hex.13978 (PMC10873686; doi:10.1111/hex.13978)
Supplement: Supplementary file 1 — Supporting information. [file HEX-27-e13978-s001.docx]

Supplement 1 - IMSA Cognitive Interview Guide

*[Welcome and Introduction]*

Thank you for agreeing to take part in this research project. My name is XX and I’m a research coordinator for this project and will be going through the interview with you today. The goal of this project is to review a survey that measures something called ‘care complexity’ and see if this survey is useful to help healthcare providers care for people with RA.

*[Project Overview]*

Care complexity is when a patient has care needs in multiple areas. For example, they may have multiple health conditions, concerns about their mental health and wellness, financial or housing stressors. They may not speak English as a first language and have concerns about expressing their needs to their healthcare provider. Complex care needs may require more time to address during doctor’s appointments or even help from different kinds of professionals (for example from mental health professionals or social workers). Unfortunately, healthcare resources are limited and patients with these needs may not get the time or proper healthcare resources to address their needs and concerns. Furthermore, we have no way of knowing which patients have complex care needs and which do not. We would like to test a survey that measures care complexity called the INTERMED Self-Assessment, or IMSA that was previously developed. We want to know if the IMSA is clearly worded and useful for people with RA.

The goal for today is for us to go through the survey question by question to better understand if the questions make sense and if they seem relevant to you. We will be collecting this type of information from different people with RA and from healthcare providers. We will use this information to understand if we need to make changes to the survey for use in future.

Before I go through what we are doing, do you have any questions?

*[Outline and Instructions]*

How it will work is that I will read the question-and-answer choices out loud to you. I will let you talk aloud about what you thought about the question and how you picked your answer choices (for patients)/how you interpreted the answer choices (for providers).

Then I will ask a few questions about the question-and-answer choices and we will move onto the next question in the survey. Finally, we would like to hear your thoughts about your healthcare needs, how your healthcare team could better identify and help you with these needs in order to understand what and what questions might be missing in the survey about healthcare needs.

If anything seems unclear, tough to answer, or does not make sense, let me know. There are no right or wrong answers. The purpose is to understand if the questions are clearly written, identify areas where the survey could be improved, and to see what topics or questions might be missing, so your honest feedback is valued.

I will be taking notes and the interview will be audio recorded so we do not miss any important details.

We will take a break after 1 hour. If you need to stop at any other point, please let me know and we can pause for as long as you need.

Before we get started, do you have any questions?

*[Start digital recorder]*

For each IMSA question, the interviewer will ask the following questions:

1. Think-aloud: For each question, ask the participant:

- “What thoughts come to mind with this question?”

2. Comprehension: For each question, ask the participant:

- “What is your understanding of this question?” / “Walk me through your understanding of what the question is asking. This will help me understand if it is clearly worded.”/ “Is it clear what the question is asking?”

3. Confidence/recall for providing a response: For each question, ask the participant:

- “How did/would you come up with a response?”/ “When reading the question, what thoughts did you have as you looked through the possible responses?” / “How did you decide which answer was right for you?”
- “Walk me through your understanding of the response choices for this question. This will help me understand if they are clearly worded.”
- “Do the response choices help you provide the answer that is best for you for this question?” / “Are the response choices clear?”
- “Are there response choices that are missing?”
- “Are there any response choices you would add?” / “Are there too many or too little options to make the choice that best fits you/your situation? Or is it just right?”
- Would it be better if there were 4 vs 5 responses? Why or why not?

4. Question-specific issues to investigate:

- “What does X mean to you?”
- “Do you feel this question has been asked already?” / “Does this question seem similar to a previous question?”
- “Do you feel it challenging to switch from answering about the past to the present or present to the future in this question compared to the previous question?”
- “How do you think this question could be fixed?”

5. Relevance:

For patients:

- “Does this question address an important aspect of your life and/or healthcare?” (Ask for each question)
- “Is it important that your healthcare provider be aware of your answer to this question?” (Ask for each question)
- “How might this help your doctor better meet your healthcare needs?” (Ask for each question)

For providers:

- Does this question address an important aspect of a patients’ life and/or healthcare? (Ask for each question)
- Is it important that you are aware of a patients’ answer to this question? (Ask for each question)
- How might this help you meet the healthcare needs of your patients? (Ask for each question)

Concluding questions:

For patients:

- Care provision: In thinking about your care from the rheumatology clinic after your diagnosis with rheumatoid arthritis, what has been your experience with clinic visits and getting the care you need?
  - Have there been challenges with this?
  - What could have been done differently?
- Can you think of a time where you needed a longer clinic appointment than was typically scheduled or needed to come in sooner than your scheduled appointment?
  - What happened? Could this have been identified ahead of time? Would that have helped?
- Have you ever needed arthritis care in between visits? If so, what was the reason and how do you access this?
  - Have you ever been to the emergency room or the hospital for RA management?
- Needs identification and support:
  - How do you learn what your healthcare needs are? For example, if you needed physiotherapy or a social worker, do you figure out your own needs and ask for support, or is this determined by talking with your doctor, nurse, completing a survey etc.
    - If your doctor/healthcare team helps you access extra support, what challenges do you experience with this process (if any)?
  - Are there questions your healthcare team could ask you to better identify your healthcare needs?
  - Do you have enough supports to manage your arthritis?
    - If not, what is missing? What else would be helpful?

- Ancillary challenges:
  - What types of things in your life cause challenges for you in terms of how you manage with your arthritis? Probes: at home/family, at work, cost of living, insurance coverage, travel (urban/rural), other health conditions, mental health status/stressors?
    - Cultural/religious/language issues/concerns?
    - Challenges with understanding/acting on the health information you receive?
    - Positive/negative experiences with the medical system and navigating it?

- Thinking of all the questions we have discussed today and your experiences, are there any missing questions/topics in the survey that would be helpful for your doctor to know about so they can better meet your healthcare needs?

For providers:

- Care provision
  - If patients needed more appointment time than allotted or needed to be seen sooner than their regularly scheduled appointment, what were the reasons that factored into this?
  - For what reasons do patients seek care in-between appointments?
    - How is this accessed? E.g. ER, hospital, urgent care, walk ins?
- Needs identification and support
  - How do you learn about the healthcare needs of your patients?
    - Do you discuss these at their first appointment? Are these regularly discussed?
      - What are the barriers in discussing these needs? E.g. time, competing concerns, etc.
  - Are there any tools you use to determine/measure healthcare needs?
    - If not, what would be helpful?
    - What barriers do you envision in being able to integrate something like this into your practice?
  - What challenges and/or gaps exist when referring/providing extra support to your patients? E.g. social work, physio, other specialists, costs to patients, etc.
- Ancillary concerns:
  - What types of challenges come up often for patients in managing their arthritis? E.g. home/family, work, insurance, comorbid conditions, mental health, costs of living etc.
- Thinking of all the questions we have discussed today and your experiences, are there any missing questions/topics in the survey that would be helpful for you to know about so you/your team can better meet your patients’ healthcare needs?

*[Wrap-up]*

This brings us to the end of the interview. Is there anything you would like to add to what’s been said?

Would you be interested in taking another look at this survey once we make changes to it?

*[Conclusion]*

Thank you so much for making the time to be interviewed and for all your thoughtful contributions.

If you have any questions or concerns, please be in touch with XX, Research Coordinator (email provided).

Supplement 2 – Summary of IMSA Cognitive Debriefing Interview Results

|  | |
| --- | --- |
| Original Survey Item | Summary of Recommendations |
| PRE1 – What is your level of understanding of the language of the country you live in?   1. Native speaker 2. Good knowledge of language 3. Moderate knowledge of language 4. Poor knowledge of language | No major revisions were recommended by patients.  Healthcare providers recommended to consider rewording the item and/or directing the answer set to a specific kind of understanding (e.g. speaking, reading, writing).  Some also recommended rewording answer choice ‘A’ and to change the order of the answer set as various interpretations of the words ‘good’ or ‘moderate’ are possible. Changing the order might help better rank the answer choices according to level of understanding and lead patients to choose a more accurate answer. |
| PRE2 – Who fills in the questionnaire?   1. I will fill in the questionnaire myself 2. I will fill in the questionnaire myself aided by someone else (a. partner/family member/friend, b. nurse, c. researcher, d. other (please specify): _______) 3. Someone else will fill in the questionnaire after consulting me (a. partner/family member/friend, b. nurse, c. researcher, d. other (please specify): _______) | No major revisions were recommended by patients.  Healthcare providers recommended switching the order of PRE1 and PRE2 to have the IMSA begin with PRE2 as that ordering of the items seemed more logical.  It was recommended to consider changing the word ‘nurse’ in the answer choices to something more general and encompassing of the other various health professions in rheumatology. One provider discussed possibly removing this item as it was not clear to them what having this information could be helpful for. |
| Add 1 – Are you taking medications almost everyday?   1. I don’t take any regular medication 2. I do take one regular medication 3. I do take several medications   If so, how many different medications each day you take? ___________ | Patients recommended to specify what medications should be included when answering this item. They also suggested to revise the wording around ‘everyday’ and ‘each day’ so that it better fits the context of RA, as some medications are taken weekly, bi weekly, monthly, or semi-annually.  Healthcare providers felt similarly about the ‘everyday’ wording and suggested it be removed from the item or more answer choices should be added to account for medications not taken daily. Another suggestion was perhaps to simplify the item and only ask how many medications patients are on and have them state a number. |
| 1a. Did you experience any physical problem in the past 5 years?   1. No, I did not 2. Yes, I experienced physical problems but for a period shorter than 3 months 3. Yes, I did experience physical problems for a period longer than 3 months 4. Yes, in the past 5 years I have experienced several short periods with physical problems | Patients found the 5-year timeframe posed in the item was too lengthy and the timeframes posed in the answer set were too short/too rigid. Patients also were unsure of what was meant or intended by the term “physical problems”.  Similarly, healthcare providers were unsure of the intent of the item and what was to be ascertained by it. |
| 1b. Do you suffer from one or more long-lasting or chronic diseases (such as diabetes, high blood pressure, rheumatoid arthritis, lung disease, or cancer)   1. I don’t have a long-lasting or chronic disease 2. I suffer one long-lasting or chronic disease 3. I suffer several long-lasting or chronic diseases | No major revisions were recommended by patients, however, one mentioned they were unsure if they should include a condition that is in remission.  Amongst healthcare providers, defining what chronic illnesses are or possibly including common chronic diseases in the item as examples might be helpful to patients when they are answering the question. Some felt that removing answer choice A should be considered as everyone completing the survey will have rheumatoid arthritis at least. |
| 2. How difficult has it been in the past 5 years to diagnose the physical problems you experienced?   1. I did not suffer of any physical problem in the past 5 years 2. The reason for my problems was immediately clear 3. After some routine investigations the reason for my problems was identified 4. After a lot of investigations the reason for my problems was identified 5. Even though a series of investigations have been taken into effect, the origins of my problems were never diagnosed | The phrase ‘physical problem’ was undefined and patients felt it would be helpful to define or indicate which physical problems should be included.  Patients felt the timeframe in the item (5-years) was too lengthy and provided responses regarding different physical problems (other than rheumatoid arthritis) because of the timeframe. The word ‘origin’ in the final answer choice was confusing to patients as well.  Healthcare providers felt similarly about the 5-year timeframe posed in the item was too long. Some felt the phrase ‘routine investigations’ might be confusing for patients and recommended using simpler terminology such as ‘basic tests’. Some felt the final answer choice needed to be revised to include the possibility that patients may have seen multiple healthcare provider(s) to obtain a diagnosis. |
| 3. How much were you daily activities (such as job, housekeeping, hobbies, going out…) restricted by physical problems during the last week?   1. I have no, or insignificant, physical problems 2. My daily activities are not or are only mildly influenced by the physical problems that I experience 3. My daily activities are moderately influenced by physical problems 4. My daily activities are severely influenced by physical problems | No major revisions suggested by patients or healthcare providers. This is the only item in the IMSA where it was recommended to keep the item as is. |
| 4a. Do you think your doctors understand the origin of your current physical problem/s?   1. I do not have physical problems at present 2. My doctors do understand the origin of my current physical problem/s 3. My doctors understand the origin of my current physical problem/s but they have some doubts 4. My doctors have many doubts about the origin of my current physical problem/s 5. My doctors still have to find the origin of my current physical problem/s | Patients were unsure of what the term ‘origin’ meant in the item and should be defined.  Healthcare providers felt similarly about the ambiguity around the word ‘origin’ in the item. Some also felt answer choices D and E were redundant and could be combined. |
| 4b. Do you think you are receiving the appropriate treatment for your current physical problem/s?   1. I do not have physical problems at present 2. I am receiving the appropriate treatment for my current physical problem/s 3. I have some doubts about the appropriateness of the treatment for my current physical problem/s 4. I have many doubts about the appropriateness of the treatment for my current physical problem/s 5. The appropriate treatment for my current physical problem/s is still to be found | Patients were unsure how rigid the term ’treatment’ was intended to be in the item, as patients were unsure if it strictly implied RA medication, strictly pharmaceutical drugs, or if it could include other medications such as birth control, vitamins, or other lifestyle recommendations suggested by their provider(s). The ambiguity about the term ‘physical problem’ persisted here.  Healthcare providers felt the term ‘appropriate’ in the item could be replaced by ‘effective’ to better ascertain patient perspectives on their treatment plan. |
| 5. In the past 5 years, how did you cope with stressful, difficult situations?   1. Generally speaking, I have always been able to cope with stressful, difficult situations 2. Sometimes I had difficulties in coping with stressful, difficult situations, which sometimes resulted in tensions and problems with my partner, family or other people 3. I often experienced difficulties with stressful, difficult situations, which often led to tensions and problems with my partner, family or other people 4. I always experience difficulties with stressful, difficult situations. They upset me and make me tense | Patients felt the 5-year timeframe posed in the item was too broad.  Healthcare providers commented on answer choices B and C as they are worded to indicate tensions with family/partners/friends and wondered if this wording was necessary. Also they commented on how some of the answer choices were double barreled. |
| 6. In your past, have you ever had psychological problems, such as being tense, anxious, down/blue or confused?   1. No, almost never 2. Yes, however without clear influence on my daily life 3. Yes, and it influenced my daily life 4. Yes, and these problems have had or still have a long-lasting effect on my daily life | Patients found that the answer set was too narrow and broadening it could allow them to find an answer choice that fit them and their personal situation better. The timeframe of the item was vague and it was unclear to patients how far to refer back to.  Healthcare providers suggested that the terms ‘tense, anxious, down/blue or confused’ should be replaced with more direct mental health concerns like anxiety and/or depression as these words may be too indirect. Providers also suggested perhaps a follow up item to this one that describes impact on daily life would be helpful from a clinical perspective and what resources to provide to patients. Some felt the term ‘psychological problems’ could be stigmatizing and the term ‘mental health’ should be used. |
| 7. Do you think it is difficult to follow your health caregivers’ recommendations?   1. No, I don’t think this is difficult 2. Yes, I think this is difficult, but I manage 3. Yes, I think this is difficult, sometimes I manage, sometimes I don’t 4. Yes, I think this is too difficult, most of the times I don’t manage | Patients did not have specific recommendations for this item.  Healthcare providers recommended the word ‘manage’ should be changed to ‘follow’ in the answer set to match the wording in the item. They also recommended the word ‘difficult’ should be specified in terms of what is implied in the context of the item – e.g. are the instructions for the medication hard to read? Is the treatment regime too complex? Are the recommendations unattainable due to financial reasons? |
| 8. At present, are you experiencing psychological problems, such as being tense, anxious, down/blue, or confused?   1. No, no problems 2. Yes, mild problems that do not affect my ability to do daily activities 3. Yes, moderate problems that affect my ability to do daily activities a little 4. d. Yes, severe problems that affect my ability to do daily activities a lot | Patients did not have specific recommendations for this item.  Healthcare providers recommended the term “psychological problems” be replaced by the term ‘mental health’ to update the language and be more current. They also recommended removing the word “confused” from the item and replacing this with other descriptors that better target feelings of anxiety/depression (e.g. unmotivated, etc.).  In terms of the answer set, healthcare providers referred to the answer choices in Q7 as a way to better capture and accommodate people who may have mental health challenges but have varying responses to them, including possibly adapting to them. Healthcare providers also preferred the answer set to Q7 to be applied to this item as to understand the impact of mental health challenges on patients, versus the answer set for this question referring to severity. |
| 9a. Do you have a job?   1. Yes 2. No | Patients recommended the item be rephrased to ask about paid employment specifically and to include a “prefer not to answer” option to be inclusive of people who may find this item intrusive/offensive.  Healthcare providers echoed similar recommendations to patients in terms of rephasing the item to directly ask about paid employment. They also suggested perhaps the item be tweaked to possibly ask if patients are engaged in part time or full-time employment. One provider suggested an open text box to learn what the patient’s occupation is as they said sometimes their occupation could impact their RA and its symptoms. |
| 9b. If you said No, please specify:   1. I am a student 2. I am retired 3. I am a housewife taking care for the household and others 4. I am disabled 5. I am more than 6-months on sick leave | Patients recommended the item be tweaked so people can check all of the options that that apply, as it was recognized that some individuals can be one or more of these situations noted in the answer set simultaneously.  Healthcare providers echoed the “check all that apply” suggestion mentioned by patients. They also recommended answer choice C be rephrased to read, “I am providing care for children and/or others in the home” to be more inclusive. They also recommended answer choice D be rephrased to better reflect language in Canadian disability policy (e.g. short term and long-term disability). Some providers suggested perhaps having an open text box to let patients elaborate on their situation if needed. |
| 9c. Have you got activities in your spare time such as volunteering, courses, sports, clubs…?   1. Yes 2. No | Some patients felt that the frequency of these activities was important to point out, so they recommended perhaps including a way have patient note which activities of theirs are regularly attended vs occasional/infrequent.  Healthcare providers recommended an open text box here to let patients elaborate on their current activities and/or activities they wish to return to which may be currently impeded by their RA and its symptoms. |
| 10. How do you generally relate to other people?   1. I have a sufficient amount of contacts with others and socialize well 2. I have contacts with others, though every now and then it might become tense 3. It is difficult for me to initiate or maintain contacts or friendships with others 4. Contacts or friendships often deteriorate into quarrels and conflicts | Patients were unsure of what this item was attempting to ascertain and recommended it be revised to make the intention of the item clearer. Some suggested it should be rephrased to be more about social support rather than relationships with others. They also felt the answer set needed to be revised to be more encompassing of different relationships and/or personality traits, e.g. people who are naturally introverted may feel slighted by the way the answer choices are worded.  Similarly, healthcare providers echoed that this item could be rephrased to be more about social support.  Both patients and healthcare providers also suggested that perhaps this item be scrapped altogether. |
| 11. Is your home living situation satisfactory? Or are adjustments needed, such as home modifications, receiving home care, or going to live somewhere else?   1. No adjustments needed, I can manage my home situation 2. No adjustments are needed, as there is enough support and care by others 3. Adjustments are needed, however not immediately 4. Immediate adjustments are needed | Patients recommended adding another answer choice that describes the immediacy of the situation and potentially the need to move immediately. One observation of note, a patient commented on how when they initially read the item, they interpreted the item to be discussing the emotional/social relationships in the home and when they read further, they realized it was discussing more physical needs and accessibility.  Healthcare providers echoed similar recommendations in that they felt the item could be reworded to ask directly about home/living arrangements, and potentially doing this by removing the first part of the item where it reads, “Is your home living situation satisfactory?” and directly asking about home modifications. Providers also recommended the answer set be reworded to follow the same pattern/scheme as previous items as a way to standardize them. |
| 12. Is assistance from your partner, family, colleagues, or friends available for you at any time?   1. I am not in need of assistance 2. Yes, assistance is available at all times 3. Yes, assistance is available but not at all times 4. The assistance I get is very limited 5. No assistance is available | Patients recommended that an answer choice be added so someone could respond that they are not in need of assistance but if it was needed, it would be available. They also recommended context around the word “assistance” in the item and whether it supposed to be interpreted as general assistance or specifically physical and/or emotional assistance.  Healthcare providers recommended the word “colleagues” be removed from the item as the item was interpreted to be more about support at home rather than at work. In addition to this, it was recommended that perhaps this item be split into 2 items to ask about assistance at home and assistance at work or other settings. It was also recommended that answer choice C should be reworded to be consistent with the others in the answer set and read, “The assistance *available to me* is very limited”. It was also suggested that the language be standardized across the answer set for this item and either use “I” language or “Yes/No” language but not both. |
| 13. Do you experience problems in getting the care you need due to living too far away, or not having any insurance, or not speaking the language very well, or differences in culture?   1. No, these are not problems for me 2. Yes, I experience some of these problems every now and then 3. Yes, I often experience some of these problems 4. Yes, some of these are big problems for me | Both patients and healthcare providers commented on this item being quadruple barrelled. Both groups recommended ways to reformat the item including, breaking up the item into several individual other items with yes/no responses for each, or listing out several situations/issues/barriers to care (with an ’other’ option to let people specify barriers to healthcare not listed) and letting people check all that apply to them and/or their situation.  Patients and providers both suggested perhaps an open text box be included to allow people to explain their circumstances.  Healthcare providers suggested the item could be reworded to read, “Do you experience problems in getting the *healthcare* you need” to better define the context of the question. |
| 14. How did you experience your contacts with doctors and healthcare providers in the last 5 years?   1. I never had problems with doctors and healthcare providers 2. I or someone close to me had negative experience(s) with doctors and healthcare providers 3. I have changed doctors and healthcare providers as a result of a negative experience 4. I frequently have changed doctors and healthcare providers because of negative experiences or lack of trust | Patients did not have specific recommendations for this item.  Healthcare providers suggested the word “doctors” be removed from the item so the item reads, “How did you experience your contacts with healthcare providers…”.  Providers recommended answer choice B have the following wording removed, “…or someone close to me” as other answer choices do to take into account other 3^rd^ party perspectives. Providers also suggested removing “lack of trust” from answer choice D as it may not be relevant or the sole reason for a negative experience. |
| 15. Who are the healthcare providers who take care for you at the moment? [multiple answers allowed]   1. I don’t receive any care 2. Primary care physician/ general practitioner 3. One medical specialist (such as: respiratory physician, cardiologist, surgeon, general physician) for physical problems 4. Several medical specialists for physical problems 5. One or more specialists for mental problems (such as: psychiatrist, psychologist, specialist for substance abuse…) 6. Social worker 7. Home nurse 8. I’m currently admitted to a hospital 9. I’m currently admitted to a psychiatric hospital 10. Other (please specify) ___________________ | Patients did not have specific recommendations for this item.  Healthcare providers had several recommendations in terms of the answer set which are listed below:  Rephrase answer choice A to instead read, “I don’t have any regular healthcare providers” because the patient would have at least one healthcare provider (rheumatologist) in rheumatology care.  Rephase answer choice C to instead read, “ One medical specialist for my physical health”.  Rephrase answer choice E to instead read, “One or more specialists for my mental health” and to consider moving answer choice F to be listed as an example of a mental health specialist in this answer choice.  Rephrase answer choice G to instead read, “Home care”.  Consider removing answer choice I regarding admission to psychiatric hospital.  Consider adding an answer choice regarding allied healthcare providers, e.g. physiotherapists, occupational therapists, etc.  Consider adding an answer choice around having a regular pharmacy/pharmacist. |
| 16. To what extent do your doctors and healthcare providers work together?   1. I do not receive care or my care is provided by just one doctor 2. My doctors and healthcare providers work together well 3. My doctors and healthcare providers work together, however sometimes more communication is needed 4. My doctors and healthcare providers do not work together quite well, leading to problems every now and then 5. My doctors and healthcare providers do not work together | Patients suggested this item would be more relevant if they were experiencing challenges with their healthcare providers not coordinating care in the way they would like, but for most, this item did not resonate.  Healthcare providers recommended the following words to be added to the item, “doctors and healthcare providers *communicate and* work together”. Providers also recommended the words “quite well” be removed from answer choice D. They also suggest context be added to answer choice D and E to specify why their providers are not working together, perhaps adding words like confusion, miscommunication, etc. |
| 17. In the next 6 months, do you expect your physical health to change? [Try to make the best estimate]   1. In the next 6 months I expect my physical health to remain the same or to get better 2. In the next 6 months I expect only a slight worsening of my physical health 3. In the next 6 months I expect a worsening of my physical health 4. d. In the next 6 months I expect a considerable worsening of my physical health | Patients recommended the timeframe in the item be shortened from 6 months to 3 months as it would be easier for patients to think about given the unpredictable nature of RA.  Healthcare providers recommended adding an answer choice of “I don’t know” or “I’m not sure”. They also recommended perhaps changing the order of the answer set to flow from better, same, to worse. Providers suggested the word “slight” in answer choice B to be better defined as it was unclear how it differs from answer choice C. The ambiguity around the term “physical health” also persisted in this item. |
| 18. In the next 6 months, do you expect your psychological well being to change? [Try to make the best estimate]   1. In the next 6 months I expect my psychological well-being to remain the same or get better 2. In the next 6 months I expect only a slight worsening of my psychological well-being 3. In the next 6 months I expect a worsening of my psychological well-being 4. In the next 6 months I expect a considerable worsening of my psychological well-being | Similarly to Q16, patients suggested to shorten the timeframe in this item.  Healthcare providers again suggested including an answer choice of “I don’t know” or “I’m not sure”. Providers also recommended replacing the term “psychological well being” to “mental health”. |
| 19. In the next 6 months do you expect that a change will be needed in the way you are currently living? [Try to make the best estimate]   1. In the next 6 months there is no need to change the way I am currently living 2. In the next 6 months I am able to stay or return to my current living situation. However homecare is needed 3. In the next 6 months a change to another living situation will be needed 4. A change to another living situation is needed immediately | Both patients and healthcare providers wondered about changes to living situations that are not just accessibility issues and perhaps considering social and/or emotional supports.  Healthcare providers suggested scraping this item in favor of Q11 that asks about current home living situation as predicting future needs might be challenging. |
| 20. In the next 6 months, do you expect that you will be in need of more help and support? [Try to make the best estimate]   1. I expect in the next 6 months that my need of care will remain the same or become less 2. I expect in the next 6 months that my need of care will increase 3. I expect in the next 6 months that my need of care will increase very much 4. I expect in the next 6 months that my need of care will increase very much and additional services will be necessary | Both patients and healthcare providers suggested defining what “help and support” is referring to in the item and if it meant around physical/accessibility or does it also include social and/or emotional help and support.  Providers also suggested possibly tweaking the answer set for consistency purposes and doing a Likert type scale going from better, worse, to same.  Providers also suggested scrapping the item as well as predicting future needs might be challenging. |
